# Consensus-based recommendations for the development and expansion of palliative day care clinics in Germany: results of a Delphi study

**DOI:** 10.1186/s12904-024-01441-3

**Published:** 2024-05-03

**Authors:** Stephanie Stiel, Alexandra Ernst, Beate Apolinarski, Hanna A. A. Röwer, Lea de Jong, Birte Burger, Sabrina Schütte, Nils Schneider, Kathrin Damm, Jona T. Stahmeyer, Franziska A. Herbst

**Affiliations:** 1 Institute for General Practice and Palliative Care, Hannover Medical School, Carl-Neuberg-Strasse 1, 30625 Hannover, Germany; 2 Center for Health Economics Research Hannover (CHERH), Leibniz University Hannover, Otto-Brenner-Strasse 7, 30159 Hannover, Germany; 3 Health Services Research Unit, AOK Niedersachsen, Hildesheimer Strasse 273, 30519 Hannover, Germany

**Keywords:** Palliative care, Day care services, Palliative day care clinics, Delphi technique, Consensus, Medical day care

## Abstract

**Background:**

Needs-based, patient-oriented palliative care includes palliative day care clinics as a specialized semi-inpatient care offer. However, the establishment and development of these facilities has been unsystematic. Research is needed to strengthen their transparency and ensure their accessibility, quality, and structural adequacy. A national Delphi study was conducted to generate appropriate recommendations for the establishment and development of palliative day care clinics in Germany.

**Methods:**

Recommendations were formulated from focus group data on the development and expansion of palliative day care clinics in Germany. Experts on in- and outpatient palliative care rated 28 recommendations for relevance and feasibility, respectively, using a 4-point Likert-type scale. Suggestions for improvement were captured via free text comments. Items were considered consented when more than 80% of the experts scored them 4 (strongly agree) or 3 (somewhat agree), regarding both relevance and feasibility.

**Results:**

A total of 23 experts (32% response rate) completed three Delphi rounds. Following the first round, 10 of 28 recommendations were revised according to participants’ comments; 1 recommendation was rejected. After the second round, 3 of these 10 recommendations were revised, while 3 were rejected. Consensus was achieved after the third round for 22 of the initial recommendations.

**Conclusions:**

The Delphi-consented recommendations provide a basis for the targeted evidence- and needs-based development of palliative day care clinics. The findings show a need for standards setting and the meaningful integration of these clinics into existing structures.

**Trial registration:**

The present study was prospectively registered on April 20, 2020, with the German Clinical Trials Register (DRKS00021446).

**Supplementary Information:**

The online version contains supplementary material available at 10.1186/s12904-024-01441-3.

## Background

Cross-sectoral in- and outpatient palliative care for patients with incurable, progressive diseases and a limited lifespan has developed significantly over the last two decades, in both general and specialized palliative care services in Germany. This development has been partly due to the socio-political relevance of palliative care, alongside demographic changes in the population. Thus, over recent years, an increasing number of palliative care units and specialized palliative home care teams have been established. In 2015, the Hospice and Palliative Care Act added a further focus on strengthening and expanding general palliative care in Germany [1]. This ongoing dynamic development aims at providing needs-based, comprehensive care in both metropolitan areas and structurally weak and rural regions, and creating equal access to palliative care for all German citizens. This overall concept of palliative care includes palliative day care clinics as specialized semi-inpatient care for outpatients [2, 3].

Internationally, the goal of palliative day care clinics is to promote autonomy and improve quality of life for patients in need of palliative care, through medical care (e.g., ascites punctures, wound management), symptom control, physiotherapy, occupational therapy, art and music therapy, complementary therapies (e.g., aromatherapy, massage), and family caregiver support [4–15]. The German guidelines for palliative care for cancer patients refer to palliative day care (clinics) as a primarily medical model of care [16, 17]. In Germany, palliative day care clinics are generally affiliated with hospitals [18, 19], and they provide medical, nursing, and psychosocial treatment to patients with complex symptoms. During a visit to a palliative day care clinic, patients can access all departments and diagnostic options of the respective hospital as needed, and they may also receive interventional therapy measures without inpatient hospital admission. Specialist (e.g., oncological) parallel treatment is also possible [20].

Patients cared for in palliative day care clinics generally require the multi-professional diagnostic and therapeutic capabilities of a hospital, but not 24-hour hospital care. Common admission criteria for such clinics include the patient’s health state allowing transport to the clinic and the patient’s need for psychological support, family caregiver relief, monitoring, and symptom control. While no positive effect of palliative day care clinics on quality of life or symptom control has been clearly demonstrated [21], qualitative studies have shown high patient satisfaction [16, 21].

In 2010, the European Association for Palliative Care estimated the need for palliative day care clinics at 1 facility per 150,000 residents [22]. A systematic study of current palliative day care clinics in Germany by the present study team in 2021 identified eight facilities, of which five offered care and three were under development or in the planning stage [18]. This number falls far short of the estimated demand.

To date, the establishment and development of palliative day care clinics in Germany has been unsystematic. Additionally, financing for these clinics has not been uniformly regulated, and thus existing palliative day care clinics are financed by different sources (i.e., statutory health insurance funds, donations, private supplementary payments, support associations) [23]. Accordingly, there is a risk of isolated solutions that are not coordinated—either with each other or with existing services—and therefore not adequately integrated into the overall care of patients at the end of life. To strengthen the transparency of palliative day care clinics in Germany over the long term and ensure their accessibility, quality, and structural adequacy, further research is needed.

## Methods

### Study aim

The present study aimed at systematically and empirically generating recommendations for the development and expansion of palliative day care clinics in Germany. Recommendations were formulated to optimize the care of terminally ill patients according to their needs and ensure the adequate integration of palliative day care clinics into the existing care landscape.

### Study design

The project “Improving health care for patients with terminal, progressive illnesses: Status and demand analysis for palliative day care clinics and day hospices and recommendations for health care planning” (ABPATITE) [24] formulated practical recommendations on the basis of empirical data gathered within the project. An online Delphi survey with palliative care experts was conducted to achieve a national consensus on these recommendations.

### Previous research steps in ABPATITE

ABPATITE was divided into three study phases [24]: Phase 1 involved a systematic survey of operating and developing palliative day care clinics and day hospices in Germany. Phase 2 consisted of three work packages aimed at determining the significance of palliative day care clinics: (a) a qualitative study of day hospices and palliative day care clinic management staff, to explore insider views of these facilities; (b) focus groups with local representatives from hospice work and palliative care, to seek the external perspectives of local care networks on the cooperating palliative care services; and (c) a quantitative survey administered to patients and their relatives on care preferences at the end of life. In phase 3, a consensus workshop (i.e., focus groups) was held to reflect on the synthesized evidence from phases 1 and 2 and to derive recommendations for the development and expansion of palliative day care clinics in Germany. The key results of the consensus workshop served as the basis for the present Delphi survey. Recommendations for day hospices were derived separately and are not addressed in this article.

### Delphi survey

#### Participants

Heterogeneous experts who were currently working with or had experience with in- and outpatient palliative and/or hospice care, including experts employed in palliative day care clinics in Germany, were invited to participate in the national Delphi survey. The group of invited experts was meant to be as heterogeneous as possible, to promote diverse perspectives on the presented recommendations. Existing contacts of the study team and the Institute for General Practice and Palliative Care at Hannover Medical School were used to recruit potential participants. In addition, relevant stakeholders were researched online and participants from previous project phases were invited (e.g., from the German Association for Palliative Medicine, the German Hospice and Palliative Care Association, home care and nursing societies, the National Association of Statutory Health Insurance Physicians, the German Medical Association, the National Association of Statutory Health Insurance Funds, the Association of Private Health Insurance Companies, and municipal branches involved in social and health policy). The experts participated voluntarily and without compensation; however, they were invited to participate in a random draw for five 50€ and ten 5€ vouchers for online stores.

#### Delphi method

The Delphi technique was employed because it enables consensus to be achieved among a wide range of knowledgeable experts when face-to-face discussion is not feasible. In total, 28 recommendations were administered anonymously online, using the software SoSci Survey V3.4 (SoSci Survey GmbH, München, Germany). The Delphi survey was developed for this study (see Supplementary 1 for Delphi survey of round 1). Participants were asked to rate each recommendation on a 4-point verbal scale (i.e., strongly agree, somewhat agree, rather disagree, disagree), according to its: (a) relevance and (b) feasibility for the development and expansion of palliative day care clinics in Germany. Experts also had the option to abstain from rating individual items by selecting a “no answer” option. Each recommendation was considered consented when at least 80% of all participants who rated the item attributed it a score of 4 (i.e., strongly agree) or 3 (i.e., somewhat agree), for both relevance and feasibility. Recommendations that did not achieve consensus in a Delphi round were revised for content and/or language, according to participants’ free text comments. These recommendations were presented in the next Delphi round to all those who participated in the previous round and entered full data.

After each Delphi round was distributed, participants were reminded of their participation a maximum of two times prior to the round closure. The first two Delphi rounds were closed after 4 weeks, while the third round was closed after 2 weeks.

#### Study material

In the first Delphi round, 28 recommendations were presented in relation to the following topics: (1) the establishment of palliative day care clinics, (2) care provision (i.e., access to palliative day care clinics, integration of family caregivers, opening hours, appointment allocation, public relations), (3) professions and cooperation (i.e., occupational groups, cooperation, volunteer work), and (4) financing. In addition, sociodemographic data were gathered for participants.

#### Data analysis

Participants in each Delphi survey round confirmed their consent to participate online prior to starting the survey. The results of all Delphi survey rounds, including the sociodemographic data collected, were exported to the software packages IBM SPSS Statistics 28 (SPSS Inc., Chicago, IL, USA) and Microsoft Excel 2016 (Microsoft Corporation, Redmond, WA; USA), and analyzed descriptively. The Guidance on Conducting and REporting DElphi Studies (CREDES) in palliative care checklist [25] was used to ensure comprehensive reporting.

## Results

### Study participants

For the first Delphi round, 72 experts were invited to participate. After two reminders were sent, the survey was closed, with 36 responses. Four cases were excluded from the analysis (i.e., two due to a lack of consent to participate and data protection, two due to the submission of an incomplete survey). Thus, 32 data sets (representing 44.4% of the invited responses) were finally included.

All 32 experts from the first round were asked to participate again in the second round. Of these, 27 experts agreed. One expert was later excluded due to a lack of consent to participate. Hence, a final response rate of 26 experts (81.3%) was achieved.

The 27 participating experts from the second Delphi round were invited to participate in the third round. Of these, 23 (85.2%) ultimately completed the survey. Thus, the final response rate was 31.9% (*n* = 23/72) (see Table 1 for participants’ sociodemographic characteristics).

**Table 1:** Sociodemographic characteristics of participants in the third Delphi study round

		***n***	**%**
**No. participants**		23	100
**Sex**	Female	13	56.5
	Male	10	43.5
**Age**	31–45 years	5	21.7
	46–60 years	14	60.9
	>60 years	4	17.4
**Professional background (multiple answers possible)**	Medicine	13	56.6
	Psychology (including psychotherapy)	1	4.3
	Social sciences/humanities/public health	3	13
	Pedagogy/social work	1	4.3
	Nursing (sciences)	3	13
	Administration/economics	2	8.7
**Specialized training in palliative care**	Yes	17	73.9
	No	6	26.1
**Field of work**	Nursing, medical care	15	65.2
	(Psycho)social care	4	17.4
	Research	2	8.7
	Reimbursement authority	2	8.7
	Public administration	3	13
	Society	3	13
**Experience in palliative or hospice care**	<1 year	2	8.7
	1–3 years	1	4.3
	3–7 years	2	8.7
	7–15 years	9	39.1
	>15 years	9	39.1
**Currently/formerly employed in a palliative day care clinic or day hospice**	Yes	3	13
	No	20	87
**Currently/formerly involved in the development/establishment of a palliative day care clinic or day hospice**	Yes	7	30.4
	No	16	69.6

### Results of each Delphi round

#### First Delphi round

In the first Delphi round, 17 out of the 28 presented recommendations achieved consensus with respect to both assessment criteria (see Table 2). For the first core theme, “establishment of palliative day care clinics,” 2 of the 4 recommendations achieved consensus. For the second core theme, “care provision,” 5 of the 9 recommendations were consented. For the third core theme, “professions and cooperation,” 9 of the 12 recommendations achieved consensus. Finally, 1 of the 3 recommendations for the fourth core theme, “financing,” was consented.

**Table 2:** Recommendations for the development and expansion of palliative day care clinics (Delphi round 1)

**Recommendations for the development and expansion of palliative day care clinics (*N* = 28)**	**Agreement relevance %**	**Agreement feasibility %**	**Decision ✓ or ⨂**
**Establishment of palliative day care clinics**			
Palliative day care clinics should be established according to patients’ needs and taking into account existing regional care structures, to close care gaps.	93.8	80.0	Revised + presented in R2
The range of services offered by palliative day care clinics should be planned, taking into account regional particularities (e.g., existing care provision), to complement existing structures according to care needs.	96.9	90.6	✓
Palliative day care clinics should determine their opening hours considering regional conditions, to complement existing structures as best possible.	90.3	67.8	Revised + presented in R2
Palliative day care clinics should have the possibility of starting with a limited offer (i.e., fewer places, limited opening hours) and adapting this offer at a later stage according to actual demand.	90.7	90.0	✓
**Care provision**			
** a) Access to palliative day care clinics**			
Patients suffering from life-limiting diseases should be given the opportunity to receive semi-inpatient treatment at a palliative day care clinic as early as possible in their disease course, if they are experiencing severe symptoms.	96.7	79.3	Revised + presented in R2
Care providers (e.g., general practitioners, medical specialists, outpatient nursing care services) for terminally ill patients whose palliative care needs cannot be adequately met with outpatient care should consider co-treatment by a palliative day care clinic at an early stage and refer patients accordingly.	96.8	82.8	✓
** b) Integration of family caregivers**			
In palliative day care clinics, measures and services for family caregivers (e.g., nursing instruction, counselling, family conferences) should be offered to strengthen patients’ home care situation.	100	96.6	✓
Measures to strengthen patients’ home care situation (e.g., nursing instructions, counselling for family caregivers) should be included in palliative day care clinics’ fixed daily rate for patients received by statutory health insurance providers.	96.7	87.5	✓
** c) Opening hours**			
Palliative day care clinics should be open three to five days per week (i.e., Monday to Friday), with fixed core working times of 6–8 hours (e.g., 8 am–4 pm).	90.3	85.8	✓
Within regular opening hours, palliative day care clinics should be able to arrange the daily duration of individual patient stays.	100	79.3	Revised + presented in R2
** d) Appointment allocation**			
Within their opening hours, palliative day care clinics should be able to make appointments on short notice (e.g., by prioritizing appointments according to urgency, in order to be a point of contact for patients with acute palliative care needs [e.g., ascites puncture]).	100	70	Revised + presented in R2
Palliative day care clinics should allow for appointments for the regular co-care of patients whose care is not covered by outpatient providers, alone.	83.3	70.6	Revised + presented in R2
** e) Public relations**			
Palliative day care clinics should introduce themselves to the public (e.g., via the local press, social media, and public events).	93.6	93.6	✓
**Professions and cooperation**			
** a) Occupational groups**			
If necessary, in-house psychologists or psycho-oncologists should be consulted for the psycho(onco)logical care of patients in palliative day care clinics.	100	74.2	Revised + presented in R2
If necessary, in-house social workers should be involved in the social care (with regard to social law) of patients in palliative day care clinics.	100	90.3	✓
If necessary, in-house therapists (e.g., speech therapists, occupational therapists, physiotherapists) should be involved in the care of patients in palliative day care clinics.	96.8	80	✓
Nursing staff with specialized training in palliative care should be permanently assigned to a palliative day care clinic in accordance with the number of treatment places.	96.9	80	✓
Medical staff with an additional qualification in palliative medicine should be permanently assigned to a palliative day care clinic in accordance with the number of treatment places.	96.9	77.4	Revised + presented in R2
Palliative day care clinics should have an internal coordination office that plans patient appointments and therapies, as well as the necessary staffing.	93.5	82.7	✓
Therapists (e.g., psychologists, physiotherapists) working in a palliative day care clinic should have an additional qualification in palliative care.	90.3	60	Revised + presented in R2
** b) Cooperation**			
Palliative day care clinics should cooperate with other hospice and palliative care providers in their region and join forces in a hospice and palliative care network.	100	96.7	✓
Palliative day care clinics should introduce themselves to local care providers in the hospice and palliative care network when they are newly established, to inform these providers (in person or via public media) about their range of services and the possibilities for patient co-care.	96.7	93.3	✓
Palliative day care clinics should make structured referrals to other care providers to promote coordinated care for patients.	96.7	90	✓
Palliative day care clinics should regularly meet (e.g., two to four times per year) online or in person with other care providers in the hospice and palliative care network (e.g., to learn from and about each other, to address problems, to optimize referrals, to present case studies for “shared lessons learned”).	100	96.7	✓
** c) Volunteer work**			
Palliative day care clinics should cooperate with outpatient hospice services, so that volunteers can complement patient care in the hospital and continue to provide support in patients’ homes.	100	93.5	✓
**Financing**			
In palliative day care clinics, staff resources for the increased case management effort (i.e., coordination and organization of patient appointments, patient transport and, if necessary, volunteer work) and coordination with other care providers should be included in the funding.	96.6	66.6	Revised + presented in R2
Palliative day care clinics should be financed through fixed daily rates for semi-inpatient treatment places.	89.3	82.6	✓
Palliative day care clinics should be able to bill their care offer via the German Uniform Value Scale (similar to, e.g., authorized outpatient clinics).	44.4	37.5	⨂

R2 = Delphi round 2; ✓ consented; ⨂ not consented and deleted from the Delphi survey

Participants disagreed on 11 items. While 9 of these 11 recommendations reached consensus in terms of relevance, participants disagreed on 10 recommendations because they assessed them as unfeasible. Inability to implement the recommendations due to a lack of funding and qualified staff emerged as the main reasons for their disagreement, in the free text fields. Experts claimed that it is unfeasible to adjust clinic opening hours according to perceived demand and to arrange appointments individually and on short notice, as there is a lack of qualified staff. Also, low awareness of palliative day care clinics was mentioned as a barrier to the establishment of early co-treatment. Ten of these unconsented recommendations were revised by the study team based on participants’ free text comments. One recommendation that did not achieve consensus for either feasibility or relevance in this first Delphi round was rejected because it was perceived to contradict another recommendation that received greater agreement. The aim of the revision was to adapt the content and/or improve the comprehensibility of the unconsented recommendations. Only these revised recommendations were presented in the second Delphi round.

#### Second Delphi round

**Fig. 1:**
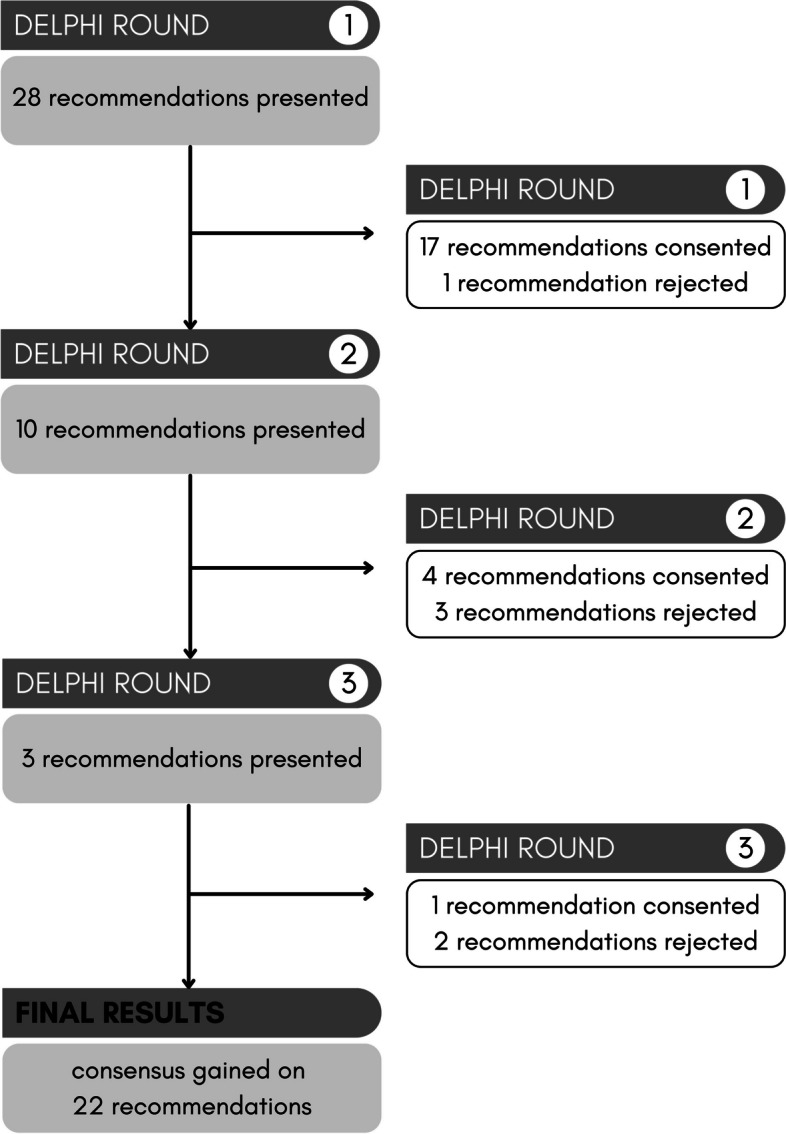
Delphi process, including the results for (non-)consented recommendations

Following the first revision cycle, a second Delphi round was carried out with the 10 revised recommendations (see Table 3 and Fig. 1). In this round, four recommendations achieved consensus (i.e., 1 from the core theme “care provision,” 2 from the core theme “professions and cooperation,” 1 from the core theme “financing”). Participants disagreed on 6 items, as they regarded them as unfeasible. Specifically, their free text comments indicated concern that palliative day clinics cannot shoulder early co-treatment on a large scale, due to a lack of facility capacities (i.e., staff, space) and the generally small number of palliative day care clinics in Germany. Furthermore, participants asked for clarification of the role of different therapeutic professions. Three recommendations were revised once again and transferred to the third Delphi round. As with the first revision, participants’ free-text comments were used to revise the recommendations. Another 3 recommendations were rejected because they contradicted other recommendations or because participants’ comments did not indicate how they could be improved. Table 3 presents detailed information on participants’ relevance and feasibility ratings in the second Delphi round.

**Table 3:** Recommendations for the development and expansion of palliative day care clinics (Delphi round 2)

**Recommendations for the development and expansion of palliative day care clinics (*N* = 10)**	**Agreement relevance %**	**Agreement feasibility %**	**Decision ✓ or ⨂ **
**Establishment of palliative day care clinics**			
Palliative day care clinics should be established considering existing regional care structures, to close care gaps and expand care offers according to patient needs.	100	70.8	⨂
Palliative day care clinics should choose their opening hours considering regional conditions (e.g., when palliative care physicians in private practice are closed), to complement existing structures as best possible.	77	48	⨂
**Care provision**			
** a) Access to palliative day care clinics**			
Patients suffering from a life-limiting illness should be given the opportunity to receive semi-inpatient treatment in a palliative day care clinic, parallel to outpatient care, as early as possible in their disease course if they are not suffering from severe symptoms.	100	65.4	Revised + presented in R3
** b) Opening hours**			
Within regular opening hours, palliative day care clinics should be able to arrange the daily duration of individual patient stays, while observing a certain minimum duration.	96	88	✓
** c) Appointment allocation**			
Within their opening hours, palliative day care clinics should hold back a small contingent of appointments, to be able to make appointments on short notice and be a point of contact for patients with acute palliative care needs (e.g., ascites puncture).	96.1	73.1	Revised + presented in R3
Palliative day care clinics should enable appointments for the regular co-care of patients whose care is not covered by outpatient providers, alone, and plan these appointments over the long term.	88.4	60	⨂
**Professions and cooperation**			
** a) Occupational groups**			
If necessary, in-house or cooperating psychologists or psycho-oncologists should be consulted for the psycho(onco)logical care of patients in palliative day care clinics.	100	84.6	✓
During opening hours, medical staff with an additional qualification in palliative medicine should be permanently assigned to a palliative day care clinic in accordance with the number of treatment places.	100	80.8	✓
Therapists (e.g., psychologists, physical therapists) working in a palliative day care clinic should be experienced in working with seriously ill patients.	100	76.9	Revised + presented in R3
**Financing**			
In palliative day care clinics, a staff member should be given time equivalents for the increased case management effort.	96.2	80	✓

R3 = Delphi round 3; ✓ consented; ⨂ not consented and deleted from the Delphi survey

#### Third Delphi round

In the third Delphi round, 3 recommendations were presented. Of these recommendations, 1 (i.e., core theme “professions and cooperation”) was consented. The other two were finally rejected, as participants still regarded them as unfeasible due to a lack of palliative day care clinic capacities.

Hence, consensus was ultimately achieved on 22 of the initial 28 recommendations. The final set included 2 recommendations on the core theme of “establishment of palliative day care clinics,” 6 recommendations addressing “care provision” (i.e., access to palliative day care clinics, integration of family caregivers, opening hours, appointment allocation, public relations), 12 addressing “professions and cooperation,” and 2 on “financing.” Table 4 provides detailed information on the 23 participants’ ratings in this round.

**Table 4:** Recommendations for the development and expansion of palliative day care clinics (Delphi round 3)

**Recommendations for the development and expansion of palliative day care clinics (*N* = 3)**	**Agreement relevance %**	**Agreement feasibility %**	**Decision ✓ or ⨂ **
**Care provision**			
**a) Access to palliative day care clinics**			
Patients suffering from life-limiting diseases and complex symptoms should be able to receive co-treatment in a palliative day care clinic at an early stage in their disease course, in parallel with their general practitioner/specialist care.	100	73.9	⨂
**b) Appointment allocation**			
Within their opening hours, palliative day care clinics should be a point of contact for patients with acute palliative care needs (e.g., ascites puncture).	100	72.8	⨂
**Professions and cooperation**			
**a) Occupational groups**			
Therapists (e.g., speech therapists, occupational therapists, physiotherapists) working in palliative day care clinics should be experienced in working with seriously ill patients.	100	82.6	✓

✓consented; ⨂ not consented and deleted from the Delphi survey

## Discussion

### Summary and discussion of the results

The present Delphi study generated 22 consented practical recommendations for the needs-based development and expansion of palliative day care clinics in Germany. Of these, 17 were immediately consented in the first round, four in the second, and one in the third. Throughout all three Delphi rounds, relevance was consistently rated as high for the great majority of the recommendations. In most cases, the major barrier to consensus was perceived unfeasibility. The consented recommendations aim at supporting the needs-based development of palliative day care clinics and the meaningful integration of such clinics into existing structures. The integration of newly established services requires cooperation between palliative and hospice care network actors, and previous studies have demonstrated that networking and collaboration between all providers in a patient’s care network promotes high-quality care [6, 26–29]. Four recommendations address the issue of cooperation with other care providers, aimed at enabling more tailored care to meet individual patient needs. The recommendations that palliative day care clinics should introduce themselves to local providers in their respective hospice and palliative care networks when they are newly established, and that information should be exchanged with other care providers on a regular basis, intend to support the interlinking of services and the smooth transition between forms of care.

Other recommendations aim at setting standards for palliative day care clinics. One recommendation calls for the involvement of therapists and another suggests the permanent assignment of nursing staff with specialized palliative care training. A third recommendation is to encourage cooperation with volunteer outpatient hospice services, to build a bridge between palliative day care and care in the patient’s home. Although these three recommendations received approval of at least 96.8% on relevance and 80% on feasibility, some participants indicated concern about their feasibility, due to a shortage of qualified staff. Several additional recommendations that received high agreement regarding relevance, did not achieve consensus for this reason.

A challenge for the implementation of the Delphi study was participants’ divided ratings and free text comments, particularly regarding recommendations for the integration of palliative day care clinic services into the existing palliative and hospice care landscape. Some participants expressed strongly that palliative day care clinics must be careful not to form a parallel structure to existing inpatient and outpatient services.

In general, the consented set of recommendations was practice-oriented, rather than political. Some participants criticized this fact, while others seemed to support it. This disagreement might be due to the fact that approximately one-third of the participants were involved in the development or establishment of a palliative day care clinic or day hospice.

### Strengths and limitations

A strength of the present study is that experts with long-standing experience in the field of palliative care in Germany participated in the three Delphi rounds. The assessments of these heterogeneous participants were—expectedly, due to their original activities—influenced by their particular interests and motivations. However, some recommendations were rejected because the free text comments contained little or no information on how they should be revised, and the study team thus had insufficient guidance on how to reformulate them.

The survey was further impacted by two technical limitations. First, the survey system apparently allowed participants to deactivate their consent to participate after having filled in the questionnaire. One recommendation, addressing the patients served by palliative day care clinics, reached 80% agreement in Delphi round 1, but the survey system assigned it an agreement value of only 79%. This is because the data of one participant, who had later deactivated their consent to participate, was included in the survey results. The study team only realized this fault when they included this one recommendation in the second Delphi round. Also, it was not possible to exclude this single participant who did not give consent for Delphi round 2. The survey system only enabled two options: (1) inviting all participants from the former round or (2) inviting all participants who had filled in the survey in the former round (even if they had deleted their consent to participate after completing the survey). This is why 27, instead of 26, experts were invited to participate in the second round.

## Conclusions

The present study generated 22 practical recommendations for the targeted, evidence- and needs-based development of palliative day care clinics in Germany. The findings show a concrete need for standards setting and the meaningful integration of these clinics into existing structures.

### Supplementary Information

Below is the link to the electronic supplementary material.

Supplementary Material 1

## Data Availability

The datasets analyzed in this study are available from the corresponding author upon reasonable request.
