# Supplementary material for: Consensus-based recommendations for the development and expansion of palliative day care clinics in Germany: results of a Delphi study
Source: BMC Palliat Care. 2024 May 3;23:116. doi: 10.1186/s12904-024-01441-3 (PMC11067173; doi:10.1186/s12904-024-01441-3)
Supplement: Supplementary file 1 — Supplementary Material 1 [file 12904_2024_1441_MOESM1_ESM.pdf]

Supplementary 1: Delphi survey of round 1

Recommendations for "Establishment of palliative day care clinics"
Please rate the following recommendations according to their relevance for the establishment and development of palliative day care clinics and their feasibility. Please use the free text field for comments and suggestions for improvement.
**Recommendation**<br>Palliative day care clinics should be established according to patients' needs and taking into account existing regional care structures, to close care gaps.
**The recommendation is relevant.**<br>☐ disagree ☐ rather disagree ☐ strongly agree ☐ somewhat agree ☐ no answer
**The recommendation is feasible.**<br>☐ disagree ☐ rather disagree ☐ strongly agree ☐ somewhat agree ☐ no answer
**Further comments and suggestions for improvement:**
**Recommendation**<br>The range of services offered by palliative day care clinics should be planned, taking into account regional particularities (e.g., existing care provision), to complement existing structures according to care needs.
**The recommendation is relevant.**<br>☐ disagree ☐ rather disagree ☐ strongly agree ☐ somewhat agree ☐ no answer
**The recommendation is feasible.**<br>☐ disagree ☐ rather disagree ☐ strongly agree ☐ somewhat agree ☐ no answer
**Further comments and suggestions for improvement:**
**Recommendation**<br>Palliative day care clinics should determine their opening hours considering regional conditions, to complement existing structures as best possible.
**The recommendation is relevant.**<br>☐ disagree ☐ rather disagree ☐ strongly agree ☐ somewhat agree ☐ no answer
**The recommendation is feasible.**<br>☐ disagree ☐ rather disagree ☐ strongly agree ☐ somewhat agree ☐ no answer
**Further comments and suggestions for improvement:**
**Recommendation**<br>Palliative day care clinics should have the possibility of starting with a limited offer (i.e., fewer places, limited opening hours) and adapting this offer at a later stage according to actual demand.
**The recommendation is relevant.**<br>☐ disagree ☐ rather disagree ☐ strongly agree ☐ somewhat agree ☐ no answer
**The recommendation is feasible.**<br>☐ disagree ☐ rather disagree ☐ strongly agree ☐ somewhat agree ☐ no answer
**Further comments and suggestions for improvement:**
**Recommendations for "Access to palliative care day clinics"**
**Background information**<br>The care provided in palliative day care clinics is aimed at terminally ill patients who wish to remain at home and whose palliative care needs cannot be adequately met in outpatient care. Palliative day care aims at delaying, avoiding, or shortening inpatient hospital stays as far as possible.

Please rate the following recommendations according to their relevance for the establishment and development of palliative day care clinics and their feasibility. Please use the free text field for comments and suggestions for improvement.
**Recommendation**<br>Patients suffering from life-limiting diseases should be given the opportunity to receive semi-inpatient treatment at a palliative day care clinic as early as possible in their disease course, if they are experiencing severe symptoms.
**The recommendation is relevant.**<br>☐ disagree ☐ rather disagree ☐ strongly agree ☐ somewhat agree ☐ no answer
**The recommendation is feasible.**<br>☐ disagree ☐ rather disagree ☐ strongly agree ☐ somewhat agree ☐ no answer
**Further comments and suggestions for improvement:**
**Recommendation**<br>Care providers (e.g., general practitioners, medical specialists, outpatient nursing care services) for terminally ill patients whose palliative care needs cannot be adequately met with outpatient care should consider co-treatment by a palliative day care clinic at an early stage and refer patients accordingly.
**The recommendation is relevant.**<br>☐ disagree ☐ rather disagree ☐ strongly agree ☐ somewhat agree ☐ no answer
**The recommendation is feasible.**<br>☐ disagree ☐ rather disagree ☐ strongly agree ☐ somewhat agree ☐ no answer
**Further comments and suggestions for improvement:**
**Recommendations for "Integration of family caregivers"**
**Background information**<br>Support for family caregivers is particularly important in the day care sector, as this can help to stabilize the patient's home care situation, enabling them to remain in their home environment for a longer period of time.
Please rate the following recommendations according to their relevance for the establishment and development of palliative day care clinics and their feasibility. Please use the free text field for comments and suggestions for improvement.
**Recommendation**<br>In palliative day care clinics, measures and services for family caregivers (e.g., nursing instruction, counselling, family conferences) should be offered to strengthen patients' home care situation.
**The recommendation is relevant.**<br>☐ disagree ☐ rather disagree ☐ strongly agree ☐ somewhat agree ☐ no answer
**The recommendation is feasible.**<br>☐ disagree ☐ rather disagree ☐ strongly agree ☐ somewhat agree ☐ no answer
**Further comments and suggestions for improvement:**
**Recommendation**<br>Measures to strengthen patients' home care situation (e.g., nursing instructions, counselling for family caregivers) should be included in palliative day care clinics' fixed daily rate for patients received by statutory health insurance providers.
**The recommendation is relevant.**<br>☐ disagree ☐ rather disagree ☐ strongly agree ☐ somewhat agree ☐ no answer
**The recommendation is feasible.**<br>☐ disagree ☐ rather disagree ☐ strongly agree ☐ somewhat agree ☐ no answer
**Further comments and suggestions for improvement:**

**Recommendations for "Opening hours"**
Please rate the following recommendations according to their relevance for the establishment and development of palliative day care clinics and their feasibility. Please use the free text field for comments and suggestions for improvement.
**Recommendation**<br>Palliative day care clinics should be open three to five days per week (i.e., Monday to Friday), with fixed core working times of 6–8 hours (e.g., 8 am–4 pm).
**The recommendation is relevant.**<br>☐ disagree ☐ rather disagree ☐ strongly agree ☐ somewhat agree ☐ no answer
**The recommendation is feasible.**<br>☐ disagree ☐ rather disagree ☐ strongly agree ☐ somewhat agree ☐ no answer
**Further comments and suggestions for improvement:**
**Recommendation**<br>Within regular opening hours, palliative day care clinics should be able to arrange the daily duration of individual patient stays.
**The recommendation is relevant.**<br>☐ disagree ☐ rather disagree ☐ strongly agree ☐ somewhat agree ☐ no answer
**The recommendation is feasible.**<br>☐ disagree ☐ rather disagree ☐ strongly agree ☐ somewhat agree ☐ no answer
**Further comments and suggestions for improvement:**
**Recommendations for "Appointment allocation"**
Please rate the following recommendations according to their relevance for the establishment and development of palliative day care clinics and their feasibility. Please use the free text field for comments and suggestions for improvement.
**Recommendation**<br>Within their opening hours, palliative day care clinics should be able to make appointments on short notice (e.g., by prioritizing appointments according to urgency, in order to be a point of contact for patients with acute palliative care needs [e.g., ascites puncture]).
**The recommendation is relevant.**<br>☐ disagree ☐ rather disagree ☐ strongly agree ☐ somewhat agree ☐ no answer
**The recommendation is feasible.**<br>☐ disagree ☐ rather disagree ☐ strongly agree ☐ somewhat agree ☐ no answer
**Further comments and suggestions for improvement:**
**Recommendation**<br>Palliative day care clinics should allow for appointments for the regular co-care of patients whose care is not covered by outpatient providers, alone.
**The recommendation is relevant.**<br>☐ disagree ☐ rather disagree ☐ strongly agree ☐ somewhat agree ☐ no answer
**The recommendation is feasible.**<br>☐ disagree ☐ rather disagree ☐ strongly agree ☐ somewhat agree ☐ no answer
**Further comments and suggestions for improvement:**
**Recommendations for "Public relations"**
Please rate the following recommendations according to their relevance for the establishment and development of palliative day care clinics and their feasibility. Please use the free text field for comments and suggestions for improvement.
**Recommendation**

Palliative day care clinics should introduce themselves to the public (e.g., via the local press, social media, and public events).
**The recommendation is relevant.**<br>☐ disagree ☐ rather disagree ☐ strongly agree ☐ somewhat agree ☐ no answer
**The recommendation is feasible.**<br>☐ disagree ☐ rather disagree ☐ strongly agree ☐ somewhat agree ☐ no answer
**Further comments and suggestions for improvement:**
**Recommendations for "Occupational groups"**
**Background information**<br>The work in a palliative day care day clinic involves working together in multi-professional and interdisciplinary teams, whereby medical consultations and diagnostic measures, as well as therapeutic offers from different departments can be consulted as needed.
Please rate the following recommendations according to their relevance for the establishment and development of palliative day care clinics and their feasibility. Please use the free text field for comments and suggestions for improvement.
**Recommendation**<br>If necessary, in-house psychologists or psycho-oncologists should be consulted for the psycho(onco)logical care of patients in palliative day care clinics.
**The recommendation is relevant.**<br>☐ disagree ☐ rather disagree ☐ strongly agree ☐ somewhat agree ☐ no answer
**The recommendation is feasible.**<br>☐ disagree ☐ rather disagree ☐ strongly agree ☐ somewhat agree ☐ no answer
**Further comments and suggestions for improvement:**
**Recommendation**<br>If necessary, in-house social workers should be involved in the social care (with regard to social law) of patients in palliative day care clinics.
**The recommendation is relevant.**<br>☐ disagree ☐ rather disagree ☐ strongly agree ☐ somewhat agree ☐ no answer
**The recommendation is feasible.**<br>☐ disagree ☐ rather disagree ☐ strongly agree ☐ somewhat agree ☐ no answer
**Further comments and suggestions for improvement:**
**Recommendation**<br>If necessary, in-house therapists (e.g., speech therapists, occupational therapists, physiotherapists) should be involved in the care of patients in palliative day care clinics.
**The recommendation is relevant.**<br>☐ disagree ☐ rather disagree ☐ strongly agree ☐ somewhat agree ☐ no answer
**The recommendation is feasible.**<br>☐ disagree ☐ rather disagree ☐ strongly agree ☐ somewhat agree ☐ no answer
**Further comments and suggestions for improvement:**
**Recommendation**<br>Nursing staff with specialized training in palliative care should be permanently assigned to a palliative day care clinic in accordance with the number of treatment places.
**The recommendation is relevant.**<br>☐ disagree ☐ rather disagree ☐ strongly agree ☐ somewhat agree ☐ no answer
**The recommendation is feasible.**<br>☐ disagree ☐ rather disagree ☐ strongly agree ☐ somewhat agree ☐ no answer

**Further comments and suggestions for improvement:**
**Recommendation**<br>Medical staff with an additional qualification in palliative medicine should be permanently assigned to a palliative day care clinic in accordance with the number of treatment places.
**The recommendation is relevant.**<br>☐ disagree ☐ rather disagree ☐ strongly agree ☐ somewhat agree ☐ no answer
**The recommendation is feasible.**<br>☐ disagree ☐ rather disagree ☐ strongly agree ☐ somewhat agree ☐ no answer
**Further comments and suggestions for improvement:**
**Recommendation**<br>Palliative day care clinics should have an internal coordination office that plans patient appointments and therapies, as well as the necessary staffing.
**The recommendation is relevant.**<br>☐ disagree ☐ rather disagree ☐ strongly agree ☐ somewhat agree ☐ no answer
**The recommendation is feasible.**<br>☐ disagree ☐ rather disagree ☐ strongly agree ☐ somewhat agree ☐ no answer
**Further comments and suggestions for improvement:**
**Recommendation**<br>Therapists (e.g., psychologists, physiotherapists) working in a palliative day care clinic should have an additional qualification in palliative care.
**The recommendation is relevant.**<br>☐ disagree ☐ rather disagree ☐ strongly agree ☐ somewhat agree ☐ no answer
**The recommendation is feasible.**<br>☐ disagree ☐ rather disagree ☐ strongly agree ☐ somewhat agree ☐ no answer
**Further comments and suggestions for improvement:**
**Recommendations for “Cooperation”**
Please rate the following recommendations according to their relevance for the establishment and development of palliative day care clinics and their feasibility. Please use the free text field for comments and suggestions for improvement.
**Recommendation**<br>Palliative day care clinics should cooperate with other hospice and palliative care providers in their region and join forces in a hospice and palliative care network.
**The recommendation is relevant.**<br>☐ disagree ☐ rather disagree ☐ strongly agree ☐ somewhat agree ☐ no answer
**The recommendation is feasible.**<br>☐ disagree ☐ rather disagree ☐ strongly agree ☐ somewhat agree ☐ no answer
**Further comments and suggestions for improvement:**
**Recommendation**<br>Palliative day care clinics should introduce themselves to local care providers in the hospice and palliative care network when they are newly established, to inform these providers (in person or via public media) about their range of services and the possibilities for patient co-care.
**The recommendation is relevant.**<br>☐ disagree ☐ rather disagree ☐ strongly agree ☐ somewhat agree ☐ no answer
**The recommendation is feasible.**

☐ disagree ☐ rather disagree ☐ strongly agree ☐ somewhat agree ☐ no answer

Further comments and suggestions for improvement:

Recommendation

Palliative day care clinics should make structured referrals to other care providers to promote coordinated care for patients.

The recommendation is relevant.

☐ disagree ☐ rather disagree ☐ strongly agree ☐ somewhat agree ☐ no answer

The recommendation is feasible.

☐ disagree ☐ rather disagree ☐ strongly agree ☐ somewhat agree ☐ no answer

Further comments and suggestions for improvement:

Recommendation

Palliative day care clinics should regularly meet (e.g., two to four times per year) online or in person with other care providers in the hospice and palliative care network (e.g., to learn from and about each other, to address problems, to optimize referrals, to present case studies for "shared lessons learned").

The recommendation is relevant.

☐ disagree ☐ rather disagree ☐ strongly agree ☐ somewhat agree ☐ no answer

The recommendation is feasible.

☐ disagree ☐ rather disagree ☐ strongly agree ☐ somewhat agree ☐ no answer

Further comments and suggestions for improvement:

Recommendations for "Volunteer work"

Background information

Care provided by volunteers complements medical and nursing care, contributes to holistic care for patients and their relatives, and can also take place in the patient's home.

Please rate the following recommendations according to their relevance for the establishment and development of palliative day care clinics and their feasibility. Please use the free text field for comments and suggestions for improvement.

Recommendation

Palliative day care clinics should cooperate with outpatient hospice services, so that volunteers can complement patient care in the hospital and continue to provide support in patients' homes.

The recommendation is relevant.

☐ disagree ☐ rather disagree ☐ strongly agree ☐ somewhat agree ☐ no answer

The recommendation is feasible.

☐ disagree ☐ rather disagree ☐ strongly agree ☐ somewhat agree ☐ no answer

Further comments and suggestions for improvement:

Recommendations for "Communication with health insurances"

Please rate the following recommendations according to their relevance for the establishment and development of palliative day care clinics and their feasibility. Please use the free text field for comments and suggestions for improvement.

Recommendation

In palliative day care clinics, staff resources for the increased case management effort (i.e., coordination and organization of patient appointments, patient transport and, if necessary, volunteer work) and coordination with other care providers should be included in the funding.

The recommendation is relevant.

☐ disagree ☐ rather disagree ☐ strongly agree ☐ somewhat agree ☐ no answer

**The recommendation is feasible.**<br>☐ disagree ☐ rather disagree ☐ strongly agree ☐ somewhat agree ☐ no answer
**Further comments and suggestions for improvement:**
**Recommendations for "Financing security"**
**Background information**<br>Care offers can be billed in two ways:<br>1) In the case of daily rates, the facility receives a previously negotiated, fixed remuneration for each patient, irrespective of the individual, actual treatment effort.<br>2) The uniform assessment standard (EBM) forms the basis for the billing of outpatient services provided by social health insurance-accredited physicians and allows, among other things, the billing of individual services per patient.<br>The following recommendations are partly contradictory. We would like to ask you to evaluate the statements independently of each other.
Please rate the following recommendations according to their relevance for the establishment and development of palliative day care clinics and their feasibility. Please use the free text field for comments and suggestions for improvement.
**Recommendation**<br>Palliative day care clinics should be financed through fixed daily rates for semi-inpatient treatment places.
**The recommendation is relevant.**<br>☐ disagree ☐ rather disagree ☐ strongly agree ☐ somewhat agree ☐ no answer
**The recommendation is feasible.**<br>☐ disagree ☐ rather disagree ☐ strongly agree ☐ somewhat agree ☐ no answer
**Further comments and suggestions for improvement:**
**Recommendation**<br>Palliative day care clinics should be able to bill their care offer via the German Uniform Value Scale (similar to, e.g., authorized outpatient clinics).
**The recommendation is relevant.**<br>☐ disagree ☐ rather disagree ☐ strongly agree ☐ somewhat agree ☐ no answer
**The recommendation is feasible.**<br>☐ disagree ☐ rather disagree ☐ strongly agree ☐ somewhat agree ☐ no answer
**Further comments and suggestions for improvement:**
